# Alzheimer's Alphabet Soup: ADRD research in NHATS and NSOC

**DOI:** 10.1093/geroni/igab046.1685

**Published:** 2021-12-17

**Authors:** Loretta Anderson, Alexandra Wennberg, Frank Lin

## Abstract

There are 5.8 million Americans living with Alzheimer’s disease, but currently there is no cure or effective treatment. Alzheimer’s disease and related dementias (ADRD) are characterized by cognitive decline, but patients also have behavioral symptoms and functional decline. Understanding the gamut of risk and prognostic factors for ADRD and those associated with the task of caring for these patients is layered. The National Health and Aging Trends Study (NHATS) and the sister National Study of Caregiving (NSOC) are excellent resources to investigate layers of ADRD incidence, progression, and caregiving in the population. NHATS is a nationally representative sample of Medicare beneficiaries aged 65 and older. Since 2011, annual in-person interviews have collected data in many areas, including health, environment, wellbeing, cognition, and function. NSOC has been conducted at three timepoints corresponding with NHATS rounds and collects detailed data on caregivers, including information on care activities, caregiver burden, and caregiver wellbeing. This symposium illustrates the broad range of ADRD research questions that can be probed using NHATS/NSOC data. The session begins with a presentation the association between caregiver burden and ADRD patient cognitive outcomes. The second presentation examines the role of physical performance as a predictor of developing ADRD. The third presentation investigates the role of dual sensory impairment – both hearing and vision impairment – on dementia incidence. The session concludes with an examination of whether hearing impairment among dementia patients is associated with different caregiving needs.

